# Separate Populations of Neurons in Ventral Striatum Encode Value and Motivation

**DOI:** 10.1371/journal.pone.0064673

**Published:** 2013-05-28

**Authors:** Gregory B. Bissonette, Amanda C. Burton, Ronny N. Gentry, Brandon L. Goldstein, Taylor N. Hearn, Brian R. Barnett, Vadim Kashtelyan, Matthew R. Roesch

**Affiliations:** 1 Department of Psychology, University of Maryland, College Park, Maryland, United States of America; 2 Program in Neuroscience and Cognitive Science, University of Maryland, College Park, Maryland, United States of America; University of Chicago, United States of America

## Abstract

Neurons in the ventral striatum (VS) fire to cues that predict differently valued rewards. It is unclear whether this activity represents the value associated with the expected reward or the level of motivation induced by reward anticipation. To distinguish between the two, we trained rats on a task in which we varied value independently from motivation by manipulating the size of the reward expected on correct trials and the threat of punishment expected upon errors. We found that separate populations of neurons in VS encode expected value and motivation.

## Introduction

Behavior can be motivated by the promise of something good or the threat of something bad. Traditional ideas about ventral striatum (VS) suggest that it is critical for energizing behavior when a valued reward is at stake [1], [2]. More recently, it has been suggested that VS is critical for signaling the predicted value of reward so that prediction errors can be generated and reinforcement learning can occur [2]–[7]. Consistent with both of these theories, activity of neurons in VS is modulated by the value associated with cues that predict reward in rats [8]–[18] and monkeys [19]–[23] performing a variety of instrumental tasks, including go/nogo [8], [21], lever pressing [9]–[11], [19], [20], discrimination [13]–[15], maze running[16]–[18], and eye movement paradigms [22], [23].

Unfortunately, it is still unclear what exactly this ‘value’ signal means, not just in VS, but in several regions throughout the brain [24]–[27]. The problem is that increased activity when high value rewards are at stake might represent predicted value, but it might also reflect the degree of motivation associated with obtaining that valued outcome. Expectation of a more valued reward leads to stronger motivation, as evidenced by measures of attention, intensity of motor output, and arousal.

Value and motivation signals can be dissociated by varying expected reward and punishment associated with task performance. Punishment and reward fall on opposite sides of the value spectrum but can induce similar levels of motivation. For example, Roesch and Olson have shown that the promise of a large reward or threat of a penalty can motivate monkeys to perform better on a delayed response task [28]. Using this method, they found that neurons in orbitofrontal cortex (OFC) carried signals related to expected value, whereas activity in premotor cortex reflected the degree of motivation associated with reward- and penalty-predicting cues.

Here, we adopted a similar strategy in rats and recorded from single neurons in VS. We designed a task that would allow us to examine firing to odor cues that predicted different levels of reward and punishment independent of the subsequent motor response by presenting the odor stimulus before the movement instruction. Rats performed a spatial task in which odor cues informed rats of the size of reward expected on correct trials and the punishment that would occur after errors. Remarkably, we found that both value and motivation signals were present in VS and were encoded by different populations of neurons. Neurons that exhibited an increase in firing to reward and reward-predicting cues fired the most for large reward and least for cues predicting risk of punishment. Neurons that showed an increase in activity to cues but a decrease to reward delivery fired more strongly for cues that induced stronger motivation. Although there was substantial overlap between waveform duration and baseline firing between these two populations, these characteristics did significantly differ, suggesting that value and motivation are represented by different population of neurons in VS.

## Methods

### Subjects

Six male Long-Evans rats at 175–200 g were obtained from Charles River Labs. Rats were tested at the University of Maryland in accordance with National Institute of Health (NIH) guidelines and approved by the Institutional Animal Care and Use Committee (IACUC) at the University of Maryland, College Park (Protocol Number: R-09-37; R0-12-66).

### Surgical Procedures and Histology

Surgical procedures followed guidelines for aseptic technique. Surgery was performed under isoflurane and all efforts were made to minimize suffering, including administration of buprenorphine and Neosporin with pain relief post surgery. Electrodes were manufactured and implanted as in prior recording experiments [29]. Rats had a drivable bundle of 10, 25-µm diameter FeNiCr wires (Stablohm 675, California Fine Wire, Grover Beach, CA) chronically implanted in the left or right hemisphere dorsal to VS (n = 6; 1.6 mm anterior to bregma, + or − 1.5 mm laterally, and 4.5 mm ventral to the brain surface). Immediately prior to implantation, these wires were freshly cut with surgical scissors to extend ∼1 mm beyond the cannula and electroplated with platinum (H_2_PtCl_6_, Aldrich, Milwaukee, WI) to an impedance of ∼300 kOhms. Cephalexin (15 mg/kg p.o.) was administered twice daily for two weeks post-operatively to prevent infection.

### Behavioral Task

Recording was conducted in aluminum chambers approximately 18″ tall on each side with downward sloping walls narrowing to an area of 12″×12″ at the bottom. A central odor port was located above two adjacent fluid wells. Directional lights were located next to fluid wells. House lights were located above the panel. The odor port was connected to an air flow dilution olfactometer to allow the rapid delivery of olfactory cues. Task control was implemented via computer. Port entry and licking was monitored by disruption of photobeams.

**Figure 1:**
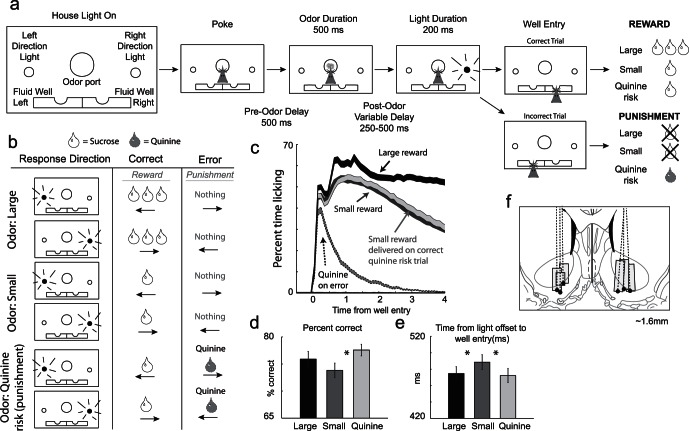
Task and behavior. A–B. House lights signaled the rat to nose poke into the center odor port and wait 500 ms before odor delivery. Two odors indicated the size (large or small) of the reward to be delivered at the end of the trial. If an error was committed on large and small reward trials, no reward was delivered. A third odor indicated that a small reward would be delivered on correct trials and that quinine would be delivered when rats responded to the wrong well. Odor presentation lasted 500 ms and was followed by a 250–500 ms post-odor variable delay, which ended with the onset of directional cue lights. Directional lights illuminated for 200 ms on either the left or right, instructing the rat to respond to the left or right fluid well, respectively. Clear drop = sucrose; gray drop = quinine. Arrow represents direction of the behavioral response. C. Average lick rate over time during recording sessions. Black = delivery of large reward; Dark gray = delivery of small reward when there was no risk; Light gray = delivery of small reward when there was a risk of quinine. Dashed gray = delivery of quinine on risk trials during which rats went to the wrong fluid well. D. Average percent correct for the three trial types. E. Average time taken to move from the odor port to the fluid well in response to the spatial cue lights. F. Locations of recording electrodes based on histology. Dashed black lines and black dots reflect the estimated center and bottom of the electrode track based on histology. Boxes represent locations were cue and reward responsive neurons were found.

The basic design of a trial is illustrated in Figure 1A and B. Rats were trained to perform a light detection task in which we varied expected size of reward and threat of punishment. The rats first learned to associate directional lights with reward locations. After the rats accurately responded to the light cues 60% of the time, they were introduced to odors that preceded the directional light. Odors indicated the size of the reward to be delivered for trials in which rats correctly responded in the direction of the light cue and the risk of punishment if the wrong well was to be selected (opposite the light cue).

Each trial began by illumination of house lights that instructed the rat to nose poke into the central odor port. Nose poking began a 500 ms pre-odor delay period. Then, one of three possible odors, which cued upcoming reward size and punishment risk, was delivered for 500 ms. Odor offset was followed by a 250–500 ms variable post-odor delay. At the end of this delay, directional lights were illuminated for 200 ms. The trial was aborted if a rat exited the odor port at any time prior to offset of a directional cue light. Left and right lights signaled which direction to make the response.

Two odors signaled that a small (1 bolus = 0.05 ml) or large (3 boli) amount of 10% sucrose solution would be available if the rat correctly responded to the direction lights. On these trials rats received nothing if they chose incorrectly. The third odor (quinine risk/punishment trials) indicated that a small reward would be delivered on correct trials, but also informed the rat that quinine (0.2 M; 0.05 ml) would be delivered on error trials. Reward and punishment were delivered immediately after entry into the well. After fluid delivery and exit from the fluid well, excess fluid was suctioned from the well and flushed with water. Odor meanings remained constant throughout the course of the experiment and were counterbalanced across rats. Odors were presented in a pseudorandom sequence such that big/small/quinine odors and left/right directional lights were presented in equal numbers (+/−1 over 250 trials). In addition, the same odor could be presented on no more than 3 consecutive trials.

### Single-unit Recording

Procedures were the same as described previously [30]. Wires were screened for activity daily; if no activity was detected, the rat was removed, and the electrode assembly was advanced 40 or 80 µm. Otherwise, active wires were selected to be recorded, a session was conducted, and the electrode was advanced at the end of the session. Neural activity was recorded using four identical Plexon Multichannel Acquisition Processor systems (Dallas, TX), interfaced with odor discrimination training chambers. The single unit signals were then sent to the Multichannel Acquisition Processor box, where they were further filtered at 250–8000 Hz, digitized at 40 kHz and amplified at 1-32X. Waveforms (>2.5∶1 signal-to-noise) were extracted from active channels and recorded to disk by an associated workstation with event timestamps from the behavior computer. Waveforms were not inverted. Duration was taken from peak to peak.

### Data Analysis

Units were sorted using Offline Sorter software from Plexon Inc. (Dallas, TX), using a template matching algorithm. Sorted files were then processed in Neuroexplorer to extract unit timestamps and relevant event markers. These data were subsequently analyzed in Matlab (Natick, MA). Baseline firing was computed during 1 second starting 2 seconds prior to odor onset. To examine activity related to odor sampling we examined activity for 500 ms starting 100 ms after odor onset (odor epoch). Note that this analysis epoch occurs while the rat is in the odor port, before onset of directional lights, and therefore, cannot be influenced by spatial cue lights or reaction time. Activity related to reward delivery was examined 1 second after reward delivery. ANOVAs, post-hoc t-tests and multiple linear regressions were used to measure differences in firing rate within and across cells (p<0.05) related to the 3 trial-types (large, small, and quinine). Pearson Chi-square tests (p<0.05) were used to compare the proportions of neurons.

## Results

Rats were trained on a task in which illumination of a left or right light indicated the location of reward (Fig. 1A and B). Prior to the spatial cue, an odor informed the rat of the size of reward and punishment that would result upon correct and incorrect performance, respectively. On two trial-types, there was no risk of punishment, just the potential of a large or small reward for a correct response. On a third trial-type, a small reward was promised for accurate performance, but there was also a risk of punishment if the rat performed the task incorrectly. The punishment was delivery of bitter quinine solution.

Rats found the quinine punishment aversive. This is illustrated in figure 1C which plots licking over time for correct and incorrect trials. Rats licked quinine immediately after its delivery on error trials but stopped abruptly soon after (Fig. 1C; dashed gray). Delivery of large reward induced the most licking which persisted for several seconds (Fig. 1C; black).

More importantly, rats showed stronger motivation on large reward and quinine risk trials. Rats were faster to move down to the fluid well in response to the lights on large reward and quinine risk trials compared to small reward trials (Fig. 1E; t-test; p’s <0.05). Rats were also more accurate on large reward and quinine risk trials (Fig. 1D). This achieved significance for punishment trials (t-test; p<0.05), but was only a trend for large reward (t-test; p = 0.07). Percent correct scores for large reward and punishment trials were not significantly different from each other (t-test; p = 0.17). Thus, overall, rats were more motivated by cues that predicted large reward and risk of punishment relative to small reward trials as shown previously in primates [28].

### Separate Populations of Neurons Encode Value and Motivation

After training, we recorded from 333 VS neurons in 6 rats (Fig. 1F). As described previously, neurons in VS showed significant increments and decrements in response to rewards and the cues that predicted them [10], [14], [17], [31], [32]. Of the 333 neurons recorded, 111 (33%) increased firing to cues during the odor epoch (baseline vs odor epoch averaged over trial-type; t-test; p<0.05). The odor epoch started 100 ms after odor onset and lasted 500 ms. This epoch consists of the time while the rat was in the odor port, before onset of the spatial cue light, which occurred no earlier than 750 ms after odor onset. Therefore, activity elicited during this analysis epoch cannot reflect the direction of the spatial light or the nature of the instrumental response.

**Figure 2:**
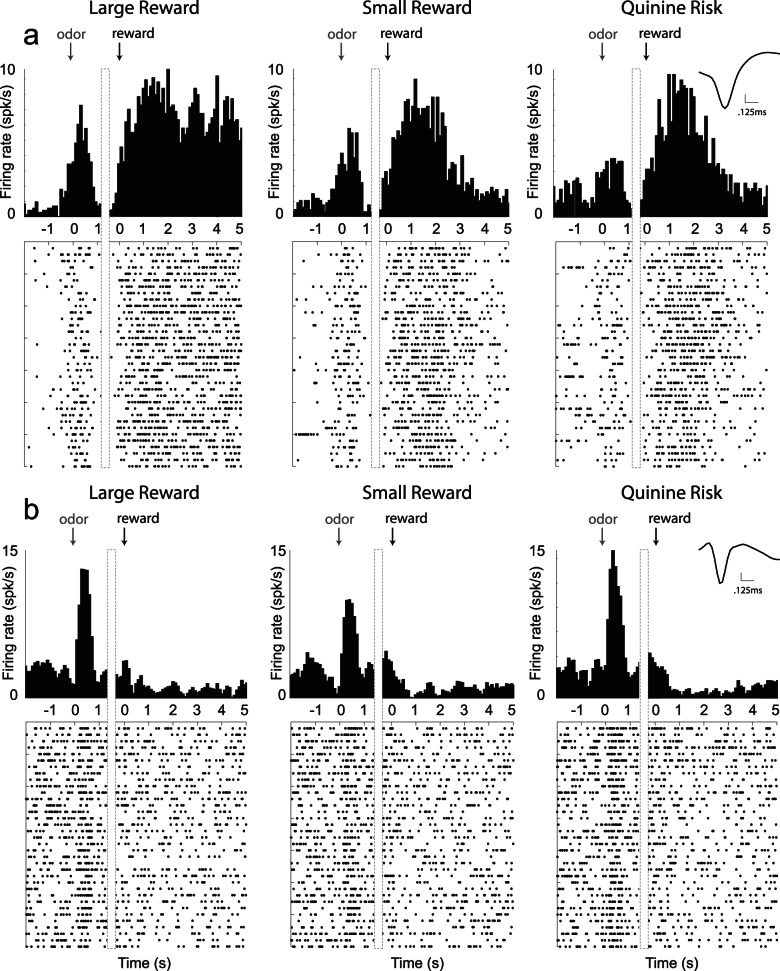
Single cell examples. Single cell examples of neurons that exhibited firing patterns consistent with value and motivation encoding on correct trials for the 3 trial-types: large reward, small reward, and punishment. Activity is aligned to odor onset (left of dashed box) and reward delivery (right of dashed box). Inset: average waveform (not inverted). A. Neuron that exhibited increased firing to odor cues and reward delivery (*increasing-type*), and was modulated by value during odor sampling. B. Neuron that increased firing to cues and decreased firing to rewards (*decreasing-type*). Activity of this neuron reflected motivation, firing stronger for large reward (left) and punishment (right) trials over small reward (middle).

Of these 111 neurons, 57(51%) and 34 (31%) also showed significant increases and decreases to reward delivery, respectively (baseline vs reward epoch averaged over trial-type; 1 s after reward; t-test; p<0.05). We will refer to these as increasing- and decreasing-type neurons. An example of the former is illustrated in figure 2A. Typical of many neurons in VS, baseline firing rate was low and increases in firing were observed during reward predicting cues and reward delivery. In addition, this neuron was significantly modulated during presentation of the odor and during delivery of different outcomes. During reward delivery, activity clearly reflected the delivery and consumption of reward, persisting for several seconds while rats consumed the reward. In addition to being modulated by reward delivery, activity of this same neuron was modulated by the expected value of the outcome predicted by the odor, firing more and less strongly for correct large reward and punishment trials, respectively, relative to small reward trials.

Remarkably, this relationship with value was mostly present in the activity of neurons that increased firing to both odor cues and reward delivery. Cue-responsive neurons that showed decreases in firing to reward delivery better reflected the motivational level associated with larger reward and risk of punishment. This type of neuron is illustrated in figure 2B. As in the other neuron, activity was stronger to odor cues that predicted large reward. However, for this neuron, activity was also strong for odor cues that predicted the risk of quinine punishment relative to small reward trials (Fig. 2B).

**Figure 3:**
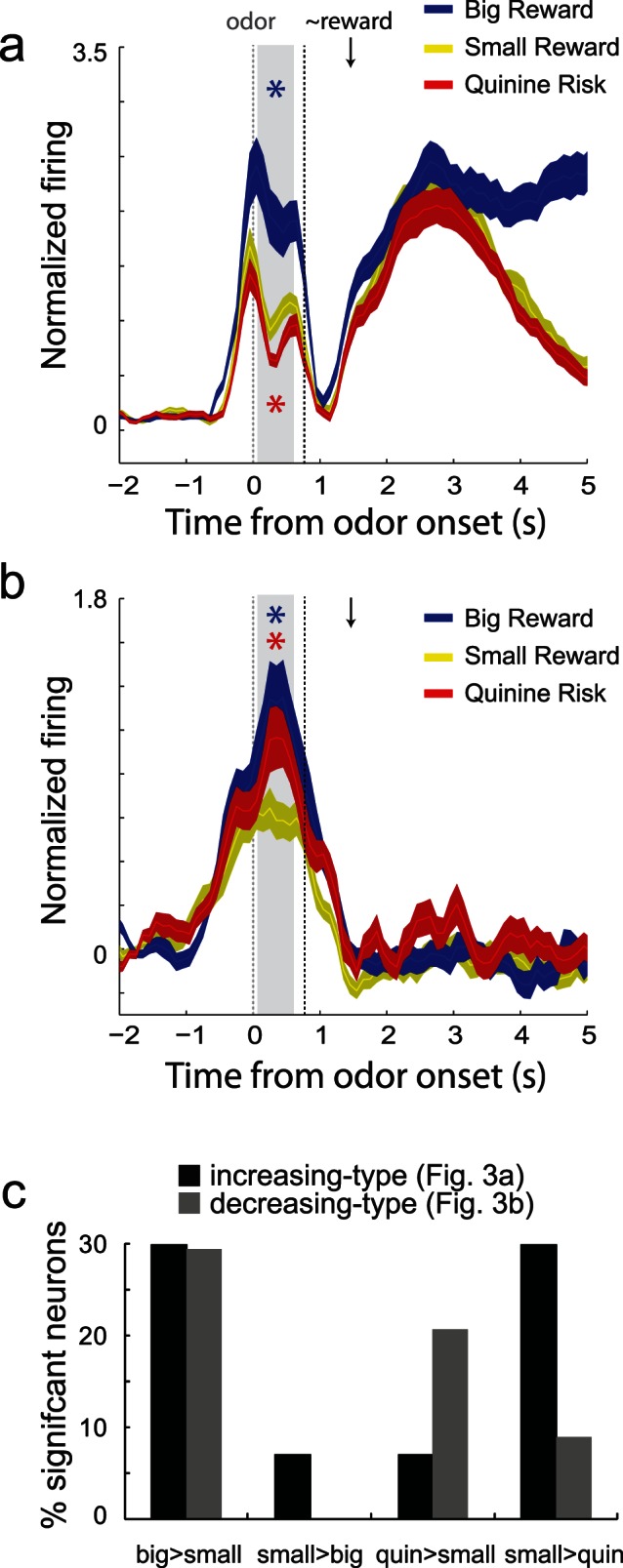
Population activity. Average normalized firing over all neurons that showed significant increases to both odor cues and reward delivery (A) and those neurons that showed significant increased and decreased firing to cues and rewards, respectively (B). Firing rates were normalized by subtracting the baseline and dividing by the standard deviation. Ribbons represent standard error of the mean (SEM). Blue asterisks indicate significant differences between average firing during the odor epoch (gray bar) between large reward and small reward trials (blue versus yellow; t-test; p<0.05). Red asterisks are the comparison between quinine punishment and small reward trials (red vs yellow; t-test; p<0.05). The odor epoch did not include time when lights were on. Gray dashed = onset of odors. Black dashed = earliest possible time lights could turn on. Black arrow marks the average time of reward delivery. C. Percentage of neurons with activity that was significantly modulated during the odor epoch (ANOVA). There was a significant difference in the frequency of neurons between increasing- and decreasing-type neurons that showed increases and decreases in firing to quinine relative to small reward trials (chi-square; p<0.05).

To quantify these effects across the two populations we made average histogram plots for both populations, averaged over all neurons. Consistent with the single cell examples, activity of neurons that fired during reward delivery reflected value (Fig. 3A), whereas activity of neurons that decreased firing during reward delivery reflected the motivational level associated with large reward and punishment trials (Fig. 3B). Differences between large reward (blue) and quinine risk (red) trials relative to small reward/no penalty trials (yellow) were significant in both populations during the odor epoch prior to onset of the spatial lights (odor epoch = 500 ms after odor onset shifted by 100 ms; gray bar; t-test; p’s <0.05). Notably, activity prior to odor onset (gray dashed line) also appeared to be selective for trial-type. This is entirely consistent with a previous report from our lab demonstrating the pre-odor activity in VS is modulated by predicted and past reward prior to odor onset [13], however, here, these differences were not significant (t-test; 200 ms prior to odor onset; p’s >0.14), likely reflecting the increased complexity of this task.

To further quantify outcome encoding during the sampling of the odors that predicted the three different outcomes, we performed an analysis to determine if activity at the single cell level during the odor epoch was significantly modulated by the three trial-types (ANOVA; p<0.05), and if the frequency of effects significantly differed between the two populations (chi-square). The result of the analysis is shown in figure 3C. A total of 42 (74%) and 20 (59%) increasing- and decreasing-type neurons showed significant modulation in the ANOVA (chi-square = 0.23; p = 0.63), respectively. In both populations, the number of neurons that showed elevated firing for large reward compared to small reward (large>small: increasing-type = 30%; decreasing-type = 29%) outnumbered those showing the opposite effect (small>large: increasing-type = 7%; decreasing-type = 0%), and the frequency of the effect did not significantly differ between the two populations (Fig. 3C; chi-square = 0.82; p = 0.37). However, as expected from firing at the population level, the frequency of neurons that were more or less active during quinine risk trials relative to small reward trials and those showing the opposite effect was significantly different between the two populations (chi-square = 5.62; p<0.05). For increasing-type neurons, 30% of neurons fired significantly more strongly (ANOVA; p<0.05) for correct small reward trials relative to quinine risk trials; only 7% showed significantly stronger firing for quinine risk trials (ANOVA; p<0.05). For decreasing-type neurons, 21% showed significantly stronger firing for quinine risk trials compared to small reward trials (ANOVA; p<0.05), whereas 9% showed the opposite effect (Fig. 3C; chi-square = 5.62; p<0.05).

These results suggest that value and motivation are encoded by different populations of neurons in VS. If this activity is important for task performance, then one might expect reduced selectivity when errors occurred. That is, failures in performance might reflect VS’s failure to signal the importance of the situation with respect to value and motivation. To address this issue we examined two different types of errors: choice errors and early unpokes. Choice errors were defined as responses made to the wrong well, which meant no reward on large and small reward trials and delivery of quinine on punishment trials. Early unpoke errors were defined as errors reflecting the impatient departure from the odor port prior to offset of the cue lights. For this type of error, house lights turned off upon port exit and no reward or punishment was delivered.

**Figure 4:**
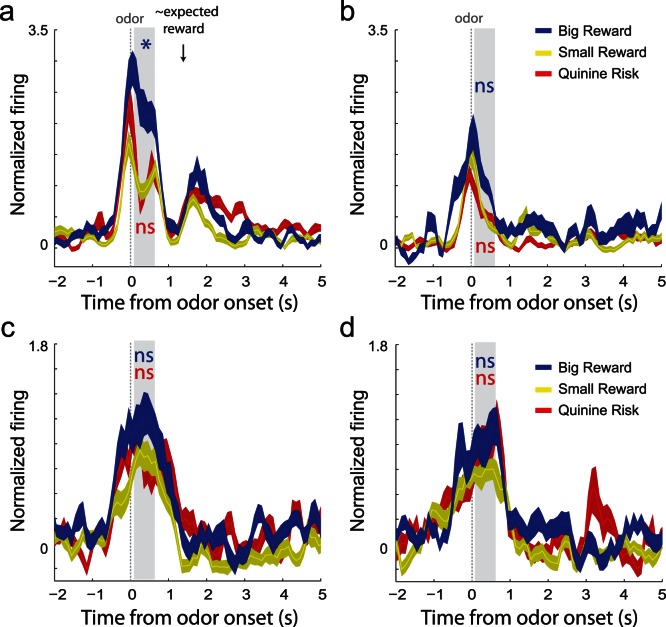
Population activity on errors. Average normalized firing for increasing-type (A–B) and decreasing-type (C–D) neurons during choice errors (left) and early unpokes (right). Firing rates are normalized by subtracting the baseline and dividing by the standard deviation. Ribbons represent standard error of the mean (SEM). Choice errors are when rats respond to the wrong well. Early unpokes are when rats exit the odor port prior to the offset of the directional cue light. Blue asterisks indicate significant differences between average firing during the odor epoch (gray bar) between large reward and small reward trials (blue versus yellow; t-test; p<0.05). Red asterisks are the comparison between quinine punishment and small reward trials (red vs yellow; t-test; p<0.05).

Figure 4 A and B plots population activity during choice errors and early unpokes for increasing-type neurons, respectively. Activity of increasing-type neurons was selective during presentation of odors on choice error trials, but only when the large reward was at stake (Fig. 4A; blue vs yellow; t-test; p<0.05). No difference was observed between incorrect small reward and punishment trials (Fig. 4A; red vs yellow; t-test; p = 0.69).

Also evident from this plot is that activity at the time when the reward would have been delivered on correct trials was elevated, suggesting that rats were expecting the reward to be delivered (Fig. 4A; ∼1 s). This is even true on trials where rats were consuming the quinine (red). Also consistent with the idea that these neurons were encoding reward expectancy, activity was significantly stronger on errors when the rats expected the larger reward. Average firing was significantly stronger 1 s after fluid well entry, during the time when reward would have been delivered on correct trials for large reward errors relative to small reward errors (blue vs yellow, t-test; p<0.05). At the single neuron level, 49% of increasing-type neurons fired significantly more strongly for large versus small reward errors and only 13% showed the opposite effect (chi-square; p<0.05). The firing pattern of these neurons is clearly in line with prediction signals needed to compute errors in reward prediction in downstream areas such as the ventral tegmental area. Notably, these differences were not present when rats committed early unpokes, likely reflecting that they were aware of their mistake as signaled by the offset of the house lights upon premature odor port exit and understood that no reward would be delivered (Fig. 4B).

Lastly, figure 4 C and D plots population firing during choice errors and early unpokes for decreasing-type neurons in VS. Unlike increasing-type neurons, none of the selectivity observed on correct trials was significant on choice error and early unpoke trials. Furthermore, these neurons did not respond to the expectation of reward (Fig. 4C; ∼1 s). These results suggest that reduced selectivity in both populations at the time of odor sampling was correlated with poor task performance, which might reflect reduced motivational drive.

### Baseline Firing and Waveform Duration

**Figure 5:**
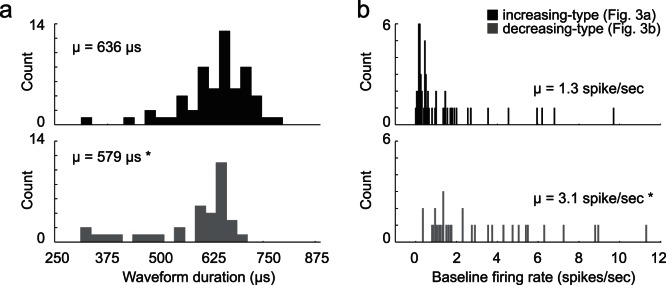
Waveform and firing characteristics. A. Distribution of waveform durations as defined by the time between two maximum amplitudes of non-inverted waveforms for increasing (top) and decreasing-type neurons (bottom). B. Distribution of average baseline firing rates taken during 1 second starting 2 seconds prior to odor onset over all trial types.

Beyond the classification of neurons based on firing being significantly modulated above and below baseline during critical task events, these neurons also presented with different waveform durations (peak to peak; waveforms not inverted) and baseline firing (1 s epoch starting 2 s before odor onset) characteristics that further distinguished them as separate populations. As illustrated in the inset of Figure 2, increasing-type neurons had broader waveforms and lower baseline firing than did decreasing-type neurons (Fig. 2A versus B inset). This was also true across the entire population. Overall, increasing-type neurons exhibited significantly wider waveforms (635 µs vs 579 µs) and lower baseline firing (1.3 vs 3.1 spikes/sec) compared to those cue-response neurons that decreased firing during reward delivery (t-test; p’s <0.05). However, this division was not entirely clear cut as illustrated by the substantial overlap in the distribution of waveform durations and baseline firing rates exhibited by these two populations (Fig. 5). With that said, it appears from this analysis that at least a subset of neurons that exhibit these different activity patterns fall into different populations.

### Correlation between Firing and Motor Output

In a final analysis we determined if cue-related activity was correlated with speed at which rats moved from the odor port to the fluid well in response to the spatial cue lights. For this analysis we performed a multiple regression analysis with reward (big, small, quinine = [1 0 0]), quinine (big, small, quinine = [0 0 1]), and response time as regressors over all odor-responsive neurons during the odor epoch (n = 111). In this analysis, we classified neurons as encoding value and motivation by determining if the slope of the correlation was the same or different for size versus quinine. Neurons that showed opposite correlations with size and quinine would be considered value-encoding, whereas neurons that exhibit correlations of the same sign would reflect a pattern of activity consistent with motivation. For example, the value-encoding neuron shown in figure 2A has a positive and negative correlation with size and quinine, respectively, whereas the motivation encoding neuron (Fig. 2B) has a positive correlation for both size and quinine.

**Figure 6:**
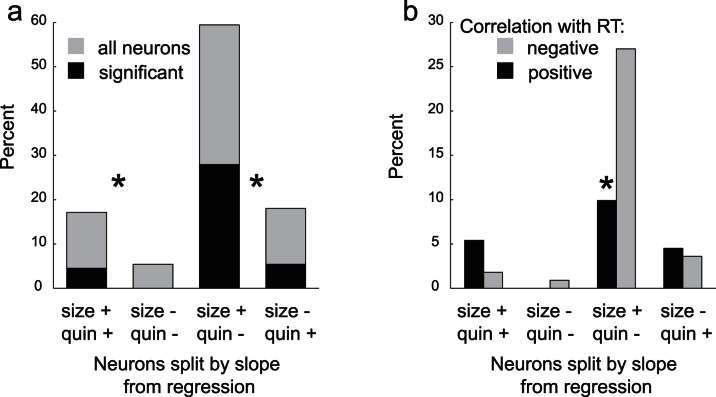
Multiple regression analysis. A. Classification of odor-responsive neurons (n = 111) into motivation and value encoding neurons based on the slope of correlations between firing rate (odor epoch), and reward size and level of quinine risk. Black bars represent neurons that showed significant correlations when adding both size and delay into the regression model. The ‘+’ and ‘−’ indicate the slope of the correlation for size and quinine. B. Percent of neurons showing a significant (p<0.05) correlation between firing rate (odor epoch) and response speed (light off to well entry) in the regression.

Consistent with the analysis described above, neurons in VS encode both value and motivation. Of those neurons that showed opposite slopes for size and quinine, the majority (59%) showed positive and negative slopes for size and quinine, respectively (Fig. 6A; size+/quin−). The correlations were significant in 47% of those neurons, which significantly outnumbered those showing the opposite effect (Fig. 6A; size−/quin+ black bars; chi-square; p<0.05). Of those neurons that exhibited slopes of the same sign, the majority showed a positive correlation with both size and quinine risk (17%; Fig. 6A; size+/quin+). Of these neurons, 26% showed significant correlations with both size and quinine, whereas none were significant for the opposite effect (Fig. 6A; size−/quin−; black bars; chi-square; p<0.05). Finally, we asked if activity in VS was correlated with the speed at which rats moved to the fluid well after illumination of the lights. Overall, 53% of neurons showed a significant correlation with response time, with 20% and 33% exhibiting positive and negative correlations (Fig. 6B). Notably, neurons that showed negative correlation with motor output tended to be value-encoding (Fig. 6B; gray; size+/quin−).

## Discussion

There has been considerable debate over which areas in the brain encode value versus other processes that vary with value [24]–[28], [33]–[45]. For example, several studies have tried to parse value from signals such as motor preparation [46], motivation [26], [28], intensity [35], [36], salience [37], [38], [45] and alertness [39]. Here, we propose that VS fulfills both evaluative and motivational functions via separate populations. Consistent with this proposal, pharmacological manipulations of VS impact motivated behaviors dependent on value expectations to guide behavior during performance of a variety of tasks [47]–[62], including reward seeking [51], cost-benefit analysis [58], [60], and delay/effort discounting [59], [63].

Two of the most prominent proposed roles of the VS in reinforcement learning and reward-guided decision-making have been its involvement as the limbic-motor interface and the ‘critic’ in actor-critic models [1]–[7], [64], [65]. As the limbic motor interface, its role is to guide decision-making by integrating value to drive motivated behavior. This model is consistent with the idea that VS serves to energize or motivate effortful behavior in response to environmental cues that carry limbic information. Here we show that VS carries information critical for such a function, with many neurons showing increased activity during the promise of a large reward and the risk of punishment.

We suggest that this signal reflects the motivational level set by cues that predict reward and punishment, which may be passed down to downstream areas more closely related to motor output. Although it is difficult to behaviorally distinguish between motivation and other processes that vary with value, such as salience, attention, arousal, and motor preparation, it appears that these neurons are truly representing motivation that serves to improve behavior. We do not think that this VS activity reflects spatial attention or motor presentation because selectivity was not present during the illumination of the lights or during the behavioral response. We also do not think that this signal simply reflects general increases in attention, arousal, or salience because it is not present when rats unexpectedly received quinine. Unexpected delivery of an aversive outcome or rewards worse than expected would increase signals related to these functions as observed in other areas, such as amygdala and anterior cingulate [66]–[72].

We also do not think that activity of these neurons reflects values associated with actions (action-value or response bias) as described previously for dorsal striatum because we are examining activity before the action is actually known, and because, in our task, value is never solely associated with one direction [22], [73]–[76]. Furthermore, in previous work, we have shown that activity in VS is more strongly associated with value of a chosen action, not the value that might be assigned to an action in a certain context or across a block of trials [77].

It might also be argued that increased firing on quinine risk trials reflects increased value of a small reward relative to receiving quinine. This would suggest that rats find situations of receiving a small reward with potential risk more valuable than situations with small reward and no risk. Thus, it follows that delivery of small reward might be construed as better when risk is actually avoided. Notably, activity during small reward delivery with and without risk was not different in either population of neurons, thus, this interpretation does not seem plausible. Furthermore, it has been shown that activity in VS neurons better reflects the sum of two presented values, representing the overall goodness of available options, during decision-making [78]. Together, this suggests that neurons with activity that exhibit elevated firing on large reward and quinine risk trials most likely reflect increased motivation associated with the situation at hand.

As for value-encoding neurons, their activity patterns appear to be more in line with VS’s proposed function as the ‘critic’ in actor-critic models. According to this model, VS signals the predicted value of reward so that downstream areas can guide decision-making and learning via errors in reward prediction [2]–[7], [64], [65]. Consistent with this idea, value encoding neurons in our dataset carried expected value signals at the time of reward delivery and during the presentation of cues that predicted different outcomes. These prediction signals were present at the same time that dopamine (DA) neurons encoded prediction errors, consistent with the idea that DA neurons use expectancy signals from VS to generate teaching signals based on errors in reward prediction [79].

Our work nicely coincides with recent imaging work in humans suggesting that out of all the ‘value-encoding’ areas in the brain, VS is one of the few that can encode value alongside other factors that vary with value, such as salience [24]. In that study authors disentangle BOLD signals related to value and salience by showing human subjects pictures of food items that ranged from being highly disliked to highly liked. Consistent with previous animal work they demonstrated that some brain areas, like OFC, encoded value, exhibiting increases from very aversive to appetitive stimuli, whereas other areas showed activations more consistent with salience; stronger activation for stimuli that elicited strong emotional responses, regardless of whether they were disliked or liked. Interestingly, signals in VS were not cleanly modulated by either, but better reflected a conglomerate of both value and salience associated with natural reactions to different types of food items. Although the subjects were not forced to eat the food, nor were they penalized for choosing incorrectly, these results suggest that processing in VS does not simply reflect value. The authors conclude that VS is modulated by salience in addition to value, but admit that they could not rule out other factors such as motivation, attention, motor preparation and arousal. Here, we show that two different populations of neurons in VS encode value and motivation.

Unfortunately, there is no perfect or ‘approved’ way to classify VS neurons based on waveform shape and firing characteristics, and attempts to do so often lead to debate and controversy. Here, we divided our neurons based on significant increases and decreases in firing rate during critical task epochs. We used this procedure because it is somewhat less arbitrary than making cutoffs based on previous papers that have classified neurons in other areas such as, dorsal striatum and the shell of nucleus accumbens. Furthermore, estimates of waveform and baseline firing differences likely vary from lab to lab based on the type of electrode used, animal preparation (species; in vivo; in vitro), epoch chosen, and classification routine. Regardless of these issues, it does appear that the majority of value and motivation encoding neurons map onto different populations [17], [80]–[87].

Most relevant to this endeavor is recent work in VS showing that neurons that increase and decrease firing to reward delivery seem to map onto putative Medium Spiny Neurons (MSNs) and Fast Spiking Interneurons (FSIs), respectively [17]. Neurons characterized as being putative FSIs generally showed an increment prior to reward delivery followed by a decrement in activity upon reward consumption, whereas putative MSNs exhibited increases in activity both before and after reward. Similarly, we found that neurons that fell into those two activity patterns showed significant differences in waveform duration and baseline firing. However, examination of the distributions of these measures clearly suggests overlap between these two populations and comparisons to averages reported in other papers suggest some mixing of the two populations [17]. Though many value-encoding neurons did show extremely low baseline firing rates (Fig. 5B; <1 spike/s) consistent with putative MSNs, the average baseline firing of motivation-encoding neurons (3.1 spikes/sec) was less than observed in this previous report (∼10 spikes/sec). This suggests that although there is no hard line dividing the two, it is likely that FSIs and MSNs both carry value and motivation signals to some degree, with MSNs being more biased toward value-encoding.

It is unclear how value and motivation signals in VS interact. However, examination of population firing suggests that after initial increments in activity in both populations, further increases and decreases in motivation-encoding neurons are accompanied by decreases and increases in firing of value-encoding neurons. Why motivation signals would have the inverse relationship with values signals during presentation of cues is an intriguing question. One possibility is that when signals related to motivating the animal are present, value signals are less crucial for guiding behavior, especially in a task that is well learned. Motivation-encoding neurons might provide feed-forward inhibition that shunts glutamatergic inputs that carry value information, thus allowing for behavior that is governed by motivated habits instead of neural representations pertaining to predicted appetitive and aversive outcomes (goal-directed). Further work is necessary to elucidate how value and motivation-encoding neurons in VS interact to influence downstream areas to guide motivated behavior [17].
